# Spurious life-threatening hypermagnesemia caused by a preanalytical error in an intensive care unit: a case report

**DOI:** 10.11613/BM.2026.031001

**Published:** 2026-08-10

**Authors:** Tara Rolić, Inja Pavlić, Ivana Sarić

**Affiliations:** 1 Faculty of Medicine, University of Osijek, Osijek, Croatia; 2 Institute of Clinical Laboratory Diagnostics, Osijek University Hospital Centre, Osijek, Croatia

**Keywords:** case report, hypermagnesemia, preanalytical error, sample re-collection, electrolyte imbalance

## Abstract

Accurate measurement of electrolytes is essential in critically ill patients. Magnesium (Mg) concentrations are particularly vulnerable to preanalytical errors, which may lead to life-threatening misinterpretation. A 57-year-old woman was admitted to the intensive care unit (ICU) due to acute decompensated heart failure and atrial fibrillation. On the second day of ICU hospitalization, serum samples analyzed in the emergency laboratory revealed critically low sodium (Na), potassium (K), and chloride (Cl) concentrations, accompanied by extremely elevated Mg concentrations that were considered incompatible with life. Given the discrepancy between life-threateningly high Mg concentrations and the patient’s stable clinical condition, as confirmed by clinicians, who reported no signs of hyporeflexia, respiratory depression, or other symptoms of Mg toxicity, the laboratory medicine professional (LMP) initiated urgent communication with the clinical staff and recommended repeating the blood draw and all analyses. A reflex glucose measurement revealed a markedly elevated concentration (55.4 mmol/L), indicating contamination with a glucose infusion. The repeated analysis showed Mg and other electrolyte concentrations within reference ranges. Further investigation confirmed that blood sampling had been performed from the same arm and venous catheter through which infusion was being administered. The abnormal laboratory findings were explained by a preanalytical error caused by blood sampling during active infusion. This case emphasizes the importance of appropriate documentation of ongoing intravenous therapy and blood sampling conditions, as well as communication between the LMP and clinical staff. Awareness of preanalytical variables is essential to prevent misinterpretation of laboratory results and inappropriate patient treatment.

## Graphical abstract

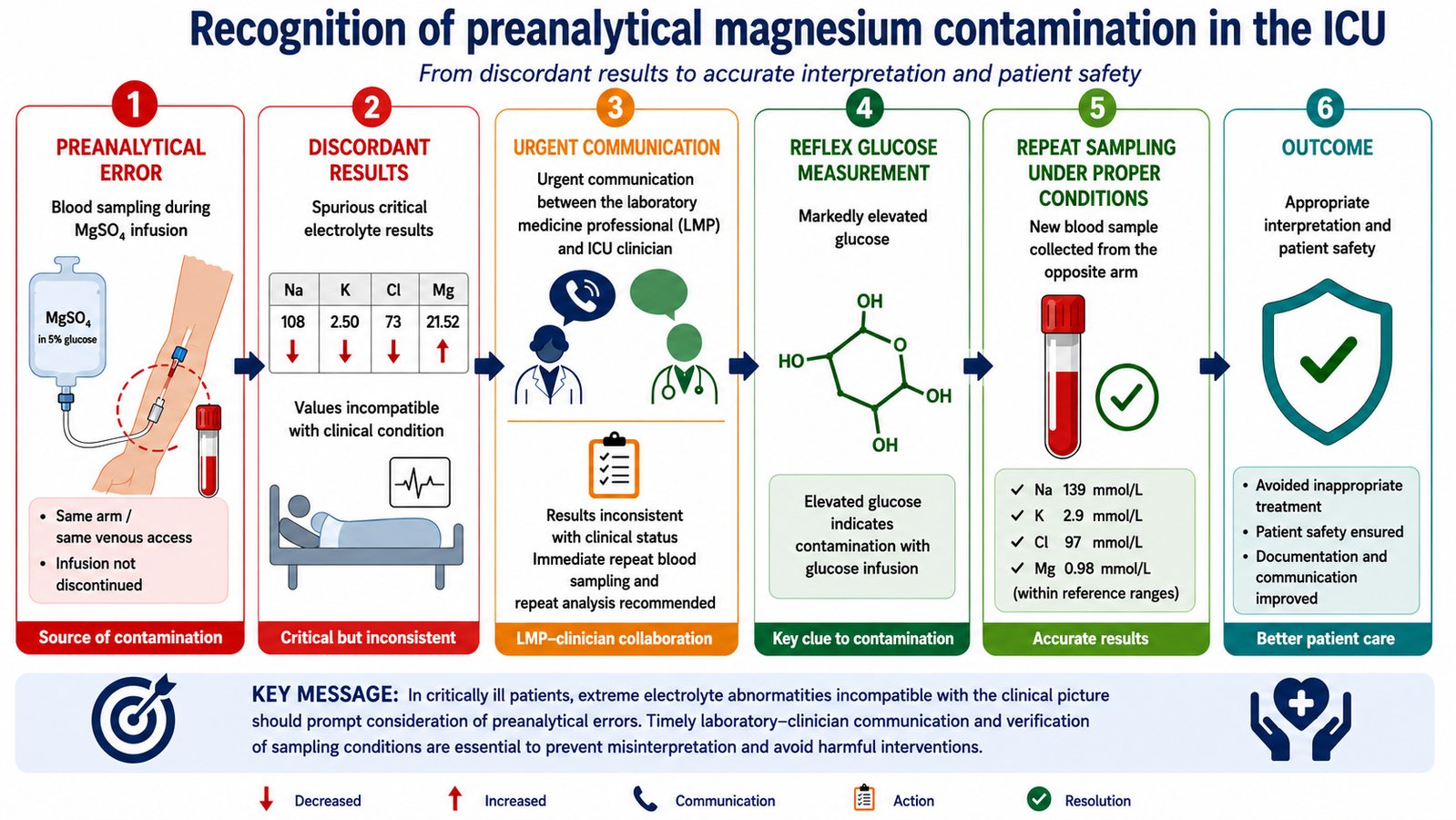

## Highlights

• Spurious hypermagnesemia from preanalytical error

• Blood drawn during ongoing magnesium infusion

• Critical values discordant with clinical condition

• Repeat sampling resolved the diagnostic dilemma

• Communication prevents inappropriate patient treatment

## Introduction

Errors in the preanalytical phase, which encompasses the whole period including test ordering, patient preparation, specimen collection, handling, transport, and processing, represent the largest source of laboratory inaccuracies and have been reported to account for over 60% of total testing errors (*1*). Such errors can lead to spurious test results that, if unrecognized, may provoke inappropriate clinical decisions or unnecessary interventions.

Accurate laboratory measurement and interpretation of serum electrolytes, including calcium (Ca) and magnesium (Mg), are essential in the management of critically ill patients, particularly those admitted to intensive care units (ICUs). Electrolyte disturbances are frequent in this population and have direct implications for hemodynamic stability, cardiac rhythm, neuromuscular function, and overall prognosis. Consequently, laboratory medicine professionals (LMP) must ensure that reported laboratory values are both analytically and clinically valid and must exercise particular vigilance when critical values are detected.

Magnesium plays a pivotal, but often underappreciated role in many physiological processes, including its role as one of the major intracellular cations, particularly in muscle and skeletal cells, as well as in cardiac conduction, vascular tone regulation, ion channel function, and numerous enzymatic processes (*2*, *3*). Hypermagnesemia, defined as a serum Mg concentration above the reference interval, is relatively uncommon and may be found in diabetic acidosis, Addison’s disease, atherosclerosis, oxalate poisoning, antacid use, and especially in chronic renal failure, or iatrogenically, through Mg-containing infusions (*4*). However, spurious hypermagnesemia may arise from preanalytical issues such as sample collection timing relative to intravenous fluid administration, sample contamination, or inadvertent inclusion of samples drawn during Mg infusion (*2*).

Given these considerations, accurate documentation of specimen collection time and clinical context is crucial; failure to do so may result in misinterpretation of laboratory results and subsequent errors in clinical interpretation and patient management.

## Case presentation

A 57-year-old female was admitted to the Osijek University Hospital Centre at the Emergency Department in January 2026 for acute decompensated heart failure with atrial fibrillation. Due to the need for continuous monitoring and advanced cardiac care, she was transferred to the cardiology ICU during the night following admission. On the following day in the ICU, a blood specimen was collected by ICU clinical staff according to routine ward procedures for evaluation of serum electrolyte status, including Ca and Mg. The sample was received in the Institute of Clinical Laboratory Diagnostics at the Department for Emergency Laboratory Medicine at 12:00 h with a request for an electrolyte panel including Ca and Mg measurements. The requisition form documented the time of sample receipt but did not clarify the exact time of phlebotomy or whether any intravenous infusions were ongoing at the time of sampling.

Laboratory analysis revealed critical elevations in Mg, which were immediately communicated to the attending clinical staff according to national recommendations for critical value reporting. All assay results are summarized in Table 1.

**Table 1:** Measured electrolyte, calcium, and magnesium concentrations in initial and repeated patient serum samples

**Analyte**	**Concentration at sample admission** **(mmol/L)** **(12.00 h)**	**Critical value**	**Reference interval (mmol/L)**	**Re-sampling** **(mmol/L)** **(13.30 h)**
Mg	21.52	> 2.00	0.65-1.05	0.98
Ca	2.38	> 3.50	2.14-2.53	2.43
Na	108	< 120	137-146	139
K	2.5	< 2.8	3.9-5.1	2.9
Cl	73	< 75	97-108	97
Mg - magnesium. Ca - calcium. Na - sodium. K - potassium. Cl - chloride.				

## Laboratory analyses

Venous blood samples were collected according to routine clinical practice and processed in accordance with national laboratory recommendations (*5*). Blood was drawn into an anticoagulant free tube, and centrifuged within the time and under the conditions recommended by the tube manufacturer. Electrolyte concentrations were measured from the primary tube on a biochemical analyzer DxC700 (Beckman Coulter, Brea, USA) using the manufacturer’s standard spectrophotometric methods for electrolyte, Ca and Mg measurements.

The first laboratory analysis revealed critically decreased concentrations of sodium, potassium and chloride, accompanied by an extremely elevated Mg concentration, inconsistent with the patient’s clinical presentation and incompatible with life. According to standard laboratory procedures, the critical results were repeated using the primary tube sample prior to communication with the clinical staff. These critical values were reported by the LMP from the emergency laboratory to the responsible ICU clinician immediately by telephone.

Given the discordance between the laboratory results and the patient’s clinical condition, the LMP initiated prompt communication with the clinical staff and requested additional information regarding the exact time of blood sampling, sampling site, and recent or ongoing intravenous therapy. Based on this phone discussion, the laboratory recommended immediate recollection of the blood sample. In addition, a reflex glucose measurement performed on the original sample revealed a markedly elevated glucose concentration (55.4 mmol/L), further supporting contamination by glucose-containing infusion fluid.

## Investigation of the preanalytical error

Further investigation revealed that blood sampling had been performed while an intravenous infusion containing 2 g magnesium sulfate (MgSO_4_) diluted in 100 mL of 5% glucose was still running. The infusion had not been discontinued prior to blood collection. The sample was collected from the same arm and venous access used for infusion administration. In addition, a delay occurred during transport of the sample to the emergency laboratory, coinciding with a clinical shift change in the ICU (*6*). The shift change did not directly influence the laboratory results; however, it contributed to delayed recognition of the preanalytical error due to incomplete transfer of information regarding ongoing infusion therapy. These circumstances resulted in contamination of the blood sample and spurious critical electrolyte results. Repeat blood sampling performed under appropriate conditions demonstrated electrolyte and Mg concentrations within the reference range, confirming the preanalytical origin of the initial abnormal results.

## Discussion

This case illustrates how preanalytical errors remain the most frequent source of laboratory testing inaccuracies, particularly in intensive care settings. In the ICU, rapid therapeutic interventions, frequent infusions, and shift changes increase the risk of incomplete information transfer and inappropriate blood sampling practices. Serum Mg measurement is especially vulnerable to such errors, as intravenous administration can cause falsely elevated serum concentrations if blood is drawn from the same or adjacent venous access site.

Unexplained, life-threatening laboratory values must always be communicated to clinicians without delay; however, critical reporting should be accompanied by immediate verification of preanalytical conditions. Key questions include: Who collected the sample? When was it collected? From which site? Was any infusion ongoing at the time of sampling? Effective communication between laboratory professionals, nurses, and clinicians is essential to rapidly identify potential sources of error and prevent inappropriate clinical interventions.

In this case, the transport delay itself did not directly affect Mg stability; however, it contributed to incomplete transfer of clinical information during ICU shift change, thereby delaying recognition of the preanalytical error.

The educational value of this case lies in reinforcing that accurate interpretation of laboratory results requires full insight into the preanalytical phase. Both junior and senior laboratory medicine professionals must remain vigilant and proactive in questioning unexpected results, while clinical staff should be continuously educated on correct sampling practices, particularly during intravenous therapy.

## Take-home message

Clear and timely communication regarding preanalytical conditions, especially the timing and site of blood sampling relative to ongoing therapy, is essential for reliable laboratory interpretation. Awareness and documentation of these factors can prevent misinterpretation of laboratory results, errors in clinical interpretation, and ultimately avoid inappropriate or harmful clinical management.

## Recommendations for laboratory practice

Always repeat blood sampling when laboratory results are incompatible with life or inconsistent with the clinical condition
Always communicate laboratory results with clinicians and nurses
Educate non-laboratory staff about the potential consequences of preanalytical errors and misinterpretation of laboratory results
Organize lectures with presentations of real cases
Pay particular attention to communication regarding ongoing therapies, recent blood sampling procedures, and critical laboratory results during clinical and laboratory shift changes

## Data Availability

The data generated and analyzed in the presented study are available from the corresponding author on request.
